# Influence of Different Carbon-Based Fillers on Electrical and Mechanical Properties of a PC/ABS Blend

**DOI:** 10.3390/polym12010029

**Published:** 2019-12-23

**Authors:** Eleonora Dal Lago, Elisabetta Cagnin, Carlo Boaretti, Martina Roso, Alessandra Lorenzetti, Michele Modesti

**Affiliations:** Department of Industrial Engineering, University of Padova, Via Marzolo, 9-35131 Padova, Italy; eleonora.dallago@phd.unipd.it (E.D.L.); elisabetta.cagnin@gmail.com (E.C.); carlo.boaretti@unipd.it (C.B.); martina.roso@unipd.it (M.R.); alessandra.lorenzetti@unipd.it (A.L.)

**Keywords:** carbon nanotubes, carbon black, graphene, conductive fillers, polymer blend

## Abstract

The present work examines the influence of different carbon-based fillers on the performance of electrically conductive polymer blend composites. More specifically, we examined and compared the effects of graphene (GR), carbon nanotubes (CNTs) and carbon black (CB) on a PC/ABS matrix by morphological investigation, electrical and physic-mechanical characterization. Electrical analyses showed volume resistivity decreased when the CNTs and CB content were increased, although the use of melt-mixed GR did not really influence this property. For the latter, solution blending was found to be more suitable to obtain better GR dispersion, and it obtained electrical percolation with a graphene content ranging from 0.5% to 1% by weight, depending on the solvent removal method that was applied. There was a gradual improvement in all of the composites’ dielectric properties, in terms of loss factor, with temperature and the concentration of the filler. As expected, the use of rigid fillers increased the composite stiffness, which is reflected in a continuous increment in the composites’ modulus of elasticity. The improvements in tensile strength and modulus were coupled with a reduction in impact strength, indicating a decrease in polymer toughness and flexibility. TEM micrographs allowed us to confirm previous results from studies on filler dispersion. According to this study and the comparison of the three carbon-based fillers, CNTs are the best filler choice in terms of electrical and mechanical performance.

## 1. Introduction

A distinctive property of all polymers is their intrinsic electrical insulating nature. Therefore, the possibility of combining their lightweight features, corrosion resistance and processability with electrical conductivity represents an attractive solution for many different applications.

A common method of providing electrical conductivity to polymers is the incorporation of conductive particles, which at specific levels of concentration and degree of dispersion create a three-dimensional network that is capable of promoting the electrical percolation of the material [1].

In recent years, the development of polymer particulate composites has become increasingly attractive due to the high economic impact of such solutions and the concurrent improvement in several material properties. Hence, the use of particles in the production of composites has become more significant in different technological fields ranging from the automotive industry (for example, as supports for metals’ electrodeposition and fuel injection systems) to the electronic sector and for electromagnetic and radio-frequency interference (EMI/RFI) shielding applications.

For electrically conductive polymer composites, the critical fraction of filler associated with the insulating–conducting transition has been variously reported and these concentration values strongly depend on factors such as the shape, size, and orientation of the filler and on the method of fabrication of the composite material [1,2,3,4].

Acrylonitrile butadiene styrene (ABS) is a widely used polymer for its easy availability, toughness, processability and luster. However, its heat distortion temperature is low and also the mechanical properties of ABS are lower than other polymers. On the other hand, polycarbonate (PC), a versatile reliable material with high impact strength, dimensional stability and toughness is a widely used polymer for high strength applications. By blending PC with ABS, properties such as heat resistance, toughness, and processability can be balanced. For these reasons, PC/ABS alloys have become one of the most important materials for fused deposition modeling processes and they are widely used in electrical, electronic, telecommunications, and automotive fields for both industrial production and personal consumption. Unfortunately, the electromagnetic emissions produced by electronic devices can interfere with other devices, causing potential problems. For this reason, many researchers have studied conductive polymer composites that show electrical conductivity and electromagnetic interference shielding effectiveness. Nanoparticle fillers are highly attractive for this purpose because they can simultaneously provide electrical conductivity and improve other properties of standard polymers, such as mechanical resistance. 

The electrical percolation limit of composites based on polymer blends can be significantly lower than the equivalent single polymer compounds, especially if the filler is localized at the interface between the two polymeric phases [5]. Also, in the case where the conductive filler is localized in one phase, a decrease in electrical percolation threshold is possible thanks to the concept of double percolation, which was originally proposed by Sumita et al. [6] for carbon black (CB) filled polymer blends. Minimizing the amount of particles needed to reach electrical conduction is necessary to preserve the mechanical properties of polymers and to reduce, as much as possible, the cost of the resulting composites. For this reason, electrically conductive polymer blends have recently gathered a lot of attention [7,8,9,10]. The possibility of coupling high performance fillers with the tailored properties provided by polymer blends creates new perspectives for applications in which simultaneous improvements in mechanical and electrical properties are critical. To such an end, a fundamental aspect is the choice of a suitable filler and the definition of the optimal processing conditions to obtain a uniform distribution and an optimal interaction between the filler and the embedding matrix.

Many previous studies have focused on the effects of different processing routes and parameters on the properties of polymer/filler composites, but only a few have investigated the differences between different fillers at the same operating conditions.

In our previous studies, we investigated the effect of processing parameters in different composite matrixes [11], on clay-based ABS nanocomposites [12,13], on PE/organoclay nanocomposites [14], on PET/PA nanocomposite blends [15] and on compatibilized polypropylene nanocomposites [16,17].

The present work examines the production of electrically conductive polymer composites based on a PC/ABS blend matrix, employing graphene (GR) [18], carbon nanotubes (CNTs) [19] and CB as fillers. This study combines morphological investigations on fillers’ dispersion with electrical and physic-mechanical characterization with the aim of investigating and comparing the effects of filler concentration/type.

## 2. Materials, Preparation and Methods 

### 2.1. Materials

The polymeric matrix selected for the production of the nanocomposites was Bayblend® T45 (Covestro AG, Leverkusen, Germany), an amorphous thermoplastic blend with a 45% w/w content of PC and a 55% w/w content of ABS which was ground to a fine powder using a Wedco single stage grinding mill and chilling the system with liquid nitrogen. The employed graphene was Avangraphene (Avanzare Innovacion Tecnologica S.L., Navarrete, Spain), which is graphene with a few layers with an average lateral size of 50–500 µm and approximate thickness of 0.7 nm. Multiwall carbon nanotubes NC7000™ (Nanocyl, Sambreville, Belgium) were used for the study. They are produced by carbon vapor deposition (CVD) and have an average diameter of 10 nm and high aspect ratio (>150) according to the producer’s data. The conductive carbon black, ENSACO® (Imerys Graphite & Carbon, Bironico, Switzerland) was employed.

### 2.2. Preparation

Melt (Brabender plastograph for preliminary analyses and then twin-screw extruder) and solution blending were employed for the preparation of the nanocomposites. The samples for the mechanical and electrical characterization, which were employed for the evaluation of the effect of the content of the different fillers in the composites, were produced according to the melt blending process. The polymer, in powder form, was dry blended with the fillers and then dried at 90 °C overnight. The composites were prepared by melt mixing using a twin-screw extruder (Collin GmbH., model ZK25, Maitenbeth, Germany) and using a melt temperature of 270 °C and a screw speed of 100 rpm, when steady flow was reached. The pellets obtained were dried at 100 °C for 8 h and then molded with a laboratory-scale injection molding machine (Canbio V55, Negri Bossi, Milan, Italy) at 250 °C for the preparation of dielectric and mechanical test samples. A Collin P200P platen press was used to prepare thin films (60 mm diameter, 0.2–0.5 mm thickness) from extruded pellets, for DC electrical measurements at 270 °C and 20 bar. 

Solution blending was employed due to the difficulties of obtaining a good GR network via melt blending (both via Brabender plastograph and via a twin-screw extruder), with the intent being to optimize GR dispersion and to obtain a low percolation threshold [20]. Samples were prepared by dissolution of PC/ABS in dimethylformamide (DMF) with a magnetic stirrer at room temperature, while simultaneously a GR suspension in DMF (1–3 mg of GR every gram of solvent) was prepared and sonicated for 15 min. The two DMF solutions were mixed in different proportions with a magnetic stirrer in order to obtain different concentrations of GR in the pristine PC/ABS matrix, i.e., 0.5, 1, 1.5, 2 and 3 wt. %. Then two different solvent removal methods were compared: evaporation and precipitation. The former method involved the evaporation of DMF at 150 °C, keeping the solution agitated. The latter required the drop-to-drop addition of 100 mL of methanol in the agitated solution to achieve precipitation of the composites, followed by filtration with 2 μm paper filter and residual solvent removal for 3 h at 80 °C under vacuum. The composites obtained by solution blending were compression molded. It was not possible to obtain injection molded samples due to the difficult preparation of large quantities of the materials.

### 2.3. Electrical and Physic-Mechanical Characterization

The electric volume resistivity was measured on the compression molded samples. For low-conductivity samples (i.e., with volume resistivity higher than 10^8^ Ω cm), a Keithley 8009 Resistivity Test Fixture combined with a Keithley electrometer Model 6517 (Cleveland, OH, USA) were used. For conductive samples (i.e., with volume resistivity lower than 10^7^ Ω cm), strips 50 mm long and 10 mm wide were connected with silver contacts to a DMM 2000 Keithley multimeter.

The dielectric analysis was performed on a TA Instruments, DEA 2970 Dielectric Analyzer (New Castle, DE, USA) at a frequency of 10^4^ Hz with injection molded samples.

The mechanical characterization was conducted using a universal testing machine (Sun 2500, Galdabini, Varese, Italy). Tensile tests were operated with a crosshead speed of 1 mm min^−1^ for modulus determination and then the speed was set to 50 mm min^−1^, according to UNI ISO 527. Flexural modulus was determined at a crosshead speed of 1.28 mm min^−1^, according to UNI ISO 178. The notched Izod impact strength was measured by an impact pendulum (Instron CEAST 9010, Norwood, MA, USA), according to UNI EN ISO 180.

The morphology of the composites was assessed by transmission electron microscopy (TEM). Slices with a thickness of about 100 nm were cut with an LKB Ultramicrotome V from injection molded samples and observed with a TEM model FEI Tecnai G12 (Hillsboro, OR, USA), 100 KV equipped with a TVIPS Tietz F114 photo camera (Gauting, Germany).

The melt flow index (MFI) test was performed according to the ASTM D 1238 standard for thermoplastics at 260 °C and with a load of 5 kg. The MFI was measured after drying the sample at 100 °C for 6 h.

## 3. Results and Discussion

### 3.1. Electrical Properties

The electrical percolation in conductive polymer composites allows the formation of a continuous network structure by the conductive filler dispersed in the polymer matrix. The lowest filler concentration in polymer matrix at which this electrical pathway is formed throughout a sample is called the percolation threshold. This means that the percolation threshold is the lowest concentration of electrically conductive filler at which a material is converted from insulating to conductive.

#### 3.1.1. Electrical Volume Resistivity Characterization

**Figure 1:**
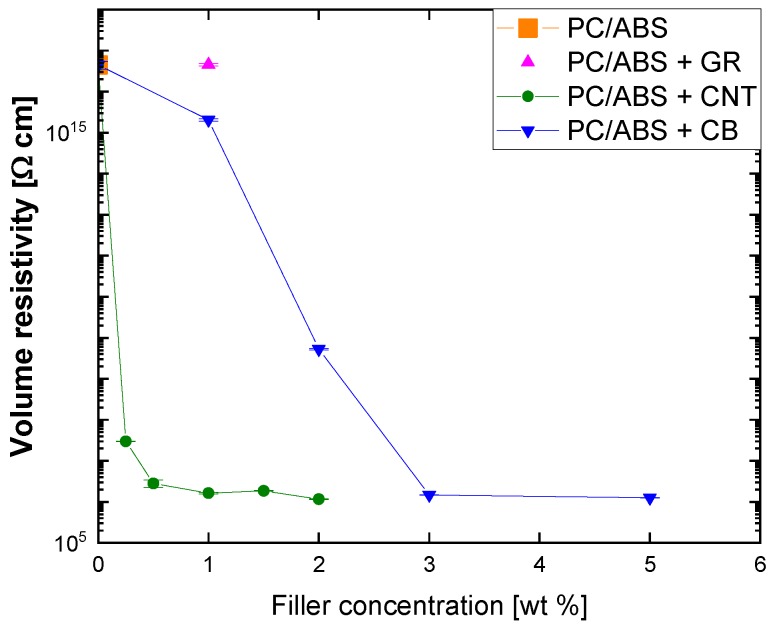
Variation in electrical resistivity with fillers’ concentration in polycarbonate/acrylonitrile butadiene styrene (PC/ABS) composites prepared by melt mixing.

The electrical resistivity characterization was carried out to determine the effect of the different fillers on the percolation threshold of the PC/ABS composites. Figure 1 shows the variation in volume resistivity of these composites as a function of the weight of the content of the fillers. As expected, the volume resistivity decreases as the percentages of CNTs and CB increase, although with significantly different behavior, while the use of GR does not really influence the resistivity when the content is 1% by weight compared to the other fillers.

The lowest percolation threshold occurred when CNTs were used compared to the other fillers employed in this study, exhibiting a power law dependence on concentration with a resistivity value of 3 × 10^7^ Ω cm at a percentage of 0.25 wt. %. Further increases in the filler content produced a reduction by an order of magnitude up to 1 wt. %, while higher loadings did not provide appreciable improvement. The samples with CB showed a slower decrease in the volume resistivity with filler loading, with a linear trend up to a percolation threshold value of 3 wt. %.

With spherical particles, as in the case of CB, the formation of a continuous path is more difficult as their diameter increases and that is the reason why, as expected, the percolation with CB was reached at a higher filler concentration compared to CNTs. On the contrary, with non-spherical particles, the filler concentration needed to obtain electrical conductivity significantly decreases with their aspect ratio [2,21]. This effect is not detectable in the samples with GR due to the inefficient mixing produced by melt compounding, which prevents good dispersion and produces a folded and deformed morphology in the filler [3], as can be seen in Section 3.3.

**Figure 2:**
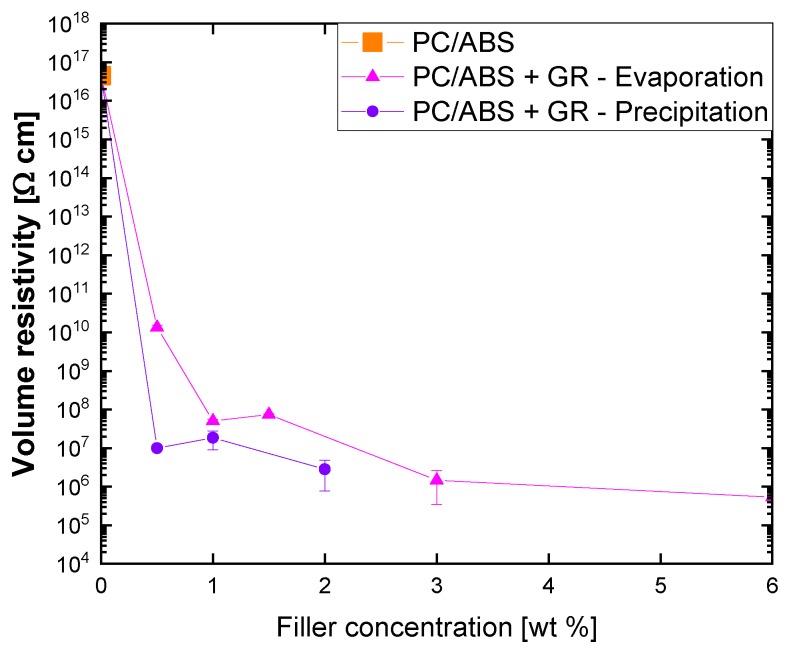
Variation of electrical resistivity with graphene (GR) concentration in PC/ABS composites prepared by solution mixing.

Since the common melt extrusion technique did not allow us to obtain good GR dispersion, to get electrical resistivity characterization of PC/ABS with the use of GR, the solution blending procedure was tested. The solution mixing allowed electrical percolation to be obtained with a GR content ranging from 0.5% to 1% by weight with solvent removal by precipitation and evaporation, respectively (Figure 2).

The evaporation method was a little slower in reaching the percolation limit because it potentially leads to filler aggregation during the slow solvent evaporation, which may be detrimental for the final composite properties [22].

The contrary results obtained for extrusion and solution dispersion of GR with regards to electrical percolation can be ascribed to the inability to provide hydrodynamic stresses high enough to overcome the agglomeration of the filler particles by melt mixing. This result, confirmed by TEM analyses, prevents the disruption of the particle agglomerates and consequently, the uniform distribution necessary to achieve percolation at low levels of filler loading. Such a conclusion is in line with the results available in the literature that suggest that, to date, melt mixing does not provide the same level of dispersion of the filler as solvent mixing methods [18].

#### 3.1.2. Dielectric Analysis

**Figure 3:**
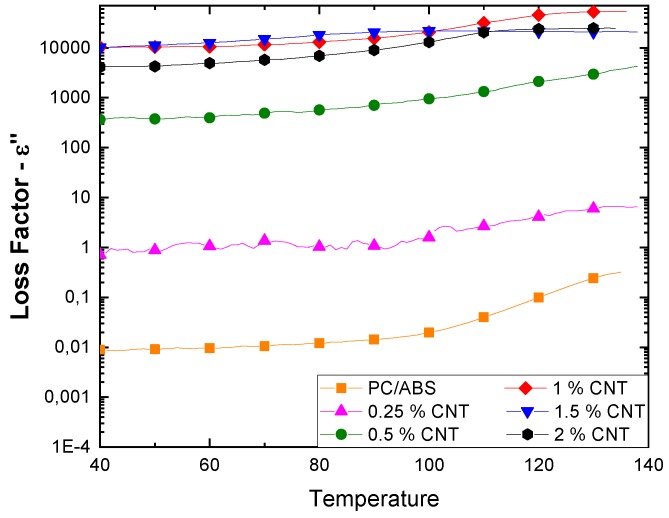
Effect of temperature on loss factor of melt-blended carbon nanotubes (CNTs)-PC/ABS composites.

Figure 3 shows how the CNTs composites’ loss factor varies with temperature depending on the filler concentration.

The graph shows the increment of ε’’ with temperature; this increment is more substantial at lower percentages of CNTs. The highest loss factor values were obtained above the percolation threshold, i.e., over 0.25 wt. %. The curves of samples with 1, 1.5 and 2 wt. % of filler are very close, almost overlapping, and their electrical resistivity values are also similar.

**Figure 4:**
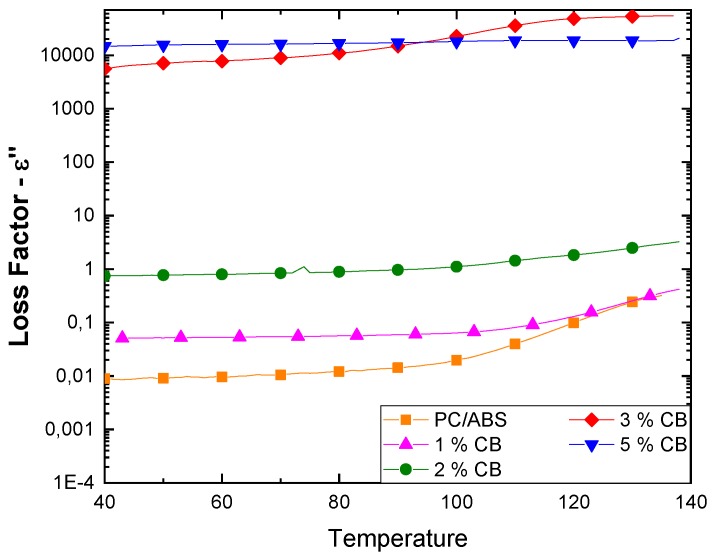
Effect of temperature on loss factor of melt blended CB-PC/ABS composites.

Figure 4 shows how the CB composites’ loss factor varies with temperature depending on the filler concentration.

As for CNTs composites, there is an increment of ε’’ with temperature; this is more significant at lower percentages of CB and the highest loss factor values were obtained above the percolation threshold, i.e., over 3 wt. %.

From these results, we observed a progressive increase in the loss factor according to the amount of filler in the composites. An abrupt increase in the loss factor was obtained with filler amounts higher than the percolation threshold, mainly due to the free flow of a large number of charge particles through the continuous conductive network that is formed. In fact, polarization becomes meaningless in the case of a conductive system where the total conductivity is governed by the DC component (ionic/electronic conductivity) as it becomes predominant [23].

The gradual improvement in the composites’ dielectric properties with temperature may be mainly ascribed to the rise in both interfacial polarization and electronic conductivity. Moreover, boosted by the higher temperature, the polymer chains have much more mobility and the formation of a conductive network is favored. In contrast, the difference in the thermal expansion in the polymer matrix (which is generally higher) and the filler has a negative influence on the dielectric properties with the temperature increase; the differential thermal expansion can disrupt chains of contacting particles, decreasing the dielectric constant. Thus, the whole effect consists of these two opposing contributions and this behavior is appreciable, especially at low filler percentages where the matrix has a fundamental role. In more highly filled composites, the nature of the rigid filler aggregates plays a key role and hence the effect of temperature is less relevant [23,24]. Near the glass transition temperature, the segmental mobility of the polymer is the dominating factor that causes an increase in the dielectric constant [25]. 

**Figure 5:**
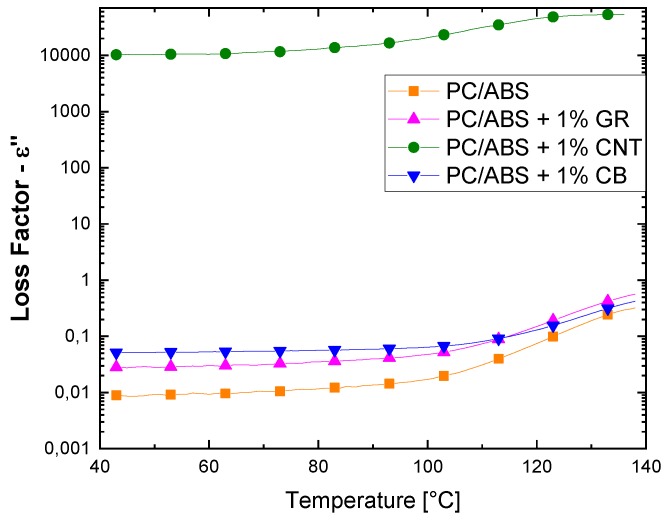
Comparison of loss factor curves of PC/ABS-based composites with 1 wt. % of filler content.

Figure 5 compares the dielectric properties of composites with 1 wt. % of different fillers. 

The 1 wt. % loss factor graph clearly shows that CNTs samples have greater conductivity. This result may be explained by the fact that at 1 wt. % only CNTs samples reached the percolation threshold, i.e., the formation of an electrical pathway through the samples because of the poor dispersion obtained with the extrusion mixing of GR and the low efficiency of spherical CB particles.

**Figure 6:**
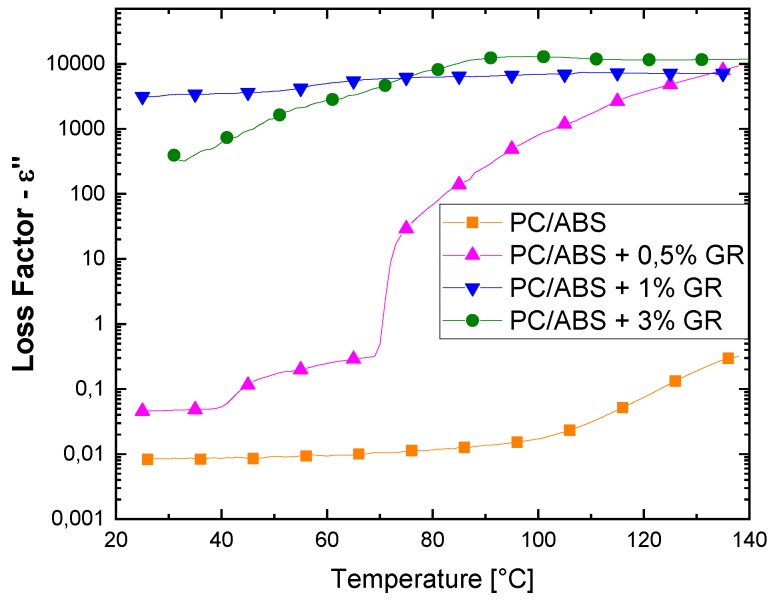
Effect of temperature on loss factor of samples obtained by solvent dispersion of GR and evaporation solvent removal method.

By analyzing the samples obtained by solvent dispersion of GR and the solvent evaporation removal method (Figure 6) it is possible to see that the loss factor is higher for composites with a filler concentration over the percolation threshold (that is, 1 and 3 wt. %). The sample with 0.5 wt. % has an anomalous curve in which the temperature effect is evident. Increases, in temperature result in high loss factor values, even when the percentage of GR is just below the percolation threshold. This is probably due to the fact that at high temperatures there is a higher dipole mobility, i.e., because this percentage is very close to the percolation limit, there is an insulating–conducting transition thanks to the high mobility of the polymer chains, which favors the formation of a conductive network.

Comparing the loss factor values of GR 1 wt. % samples obtained by extrusion and solution mixing (presented in Figure 5 and Figure 6, respectively), it is evident that the solvent method achieves better filler dispersion and thus improves the dielectric performance, thanks to the formation of a continuous network instead of coarse agglomerations. Also, the different behaviors of the dielectric properties of GR composites obtained with extrusion and solution dispersion suggest that extrusion mixing is not able to well distribute GR small sheets in these operating conditions.

### 3.2. Mechanical Properties

The enhancement of mechanical performance in polymer composites is strictly correlated to the degree of the dispersion of the fillers, their alignment within the matrix and the extent of the interfacial adhesion, which is fundamental for the formation of a reinforced composite. The latter requires that the interaction of the polymer matrix with the fillers is strong enough to support the load transfer to the particles instead of the formation of two surfaces slipping between each other [18].

#### 3.2.1. Elastic Modulus

**Figure 7:**
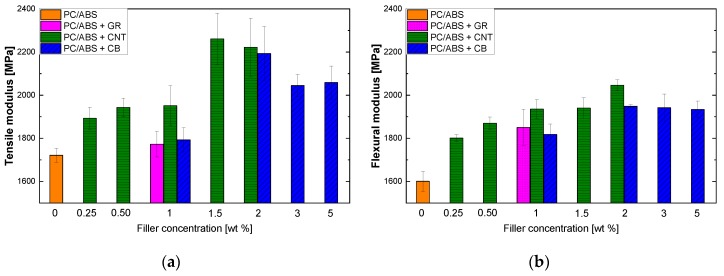
Elastic moduli of composites obtained by melt blending at different concentrations of filler: (**a**) tensile modulus; (**b**) flexural modulus.

As expected, rigid fillers increase the composite stiffness, which was reflected by a continuous increment in the composite modulus of elasticity (Figure 7). The same behavior was detected for both the tensile and flexural tests, as depicted in Figure 7a,b, respectively. 

Generally, the increase was progressive up to a 2% filler loading for both tensile and flexural modulus, with the best results displayed by the samples containing CNTs. In this case, the tensile modulus of PC/ABS increased by more than 30% (the flexural one increased by more than 20%) with just 1.5 wt. % of CNTs. Also, CB resulted in a higher elastic modulus with respect to the pristine polymer, but the improvement was moderate compared to the CNTs results due to the difference in the dimensional scale of the domain for the two particles and to their different shapes [26,27,28]. This is because Young’s modulus is affected by the amount of bonded polymer, which is in turn related to surface area and therefore, to particle size and shape. Generally, increasing the fillers’ aspect ratio results in an increase in the elastic moduli of the composite [29]. The influence of the dimensionality (1D, 2D, and 3D), that is, the aspect ratio, of the nanofiller is pronounced in the tensile properties, as previously noted by Pradhan et al. [30]. They demonstrated the following ranking of the reinforcing ability of fillers with different dimensionalities: 1D filler > 2D filler > 3D. This is in agreement with the better results obtained with CNTs, which have a 1D dimension, compared to those obtained with CB, which has 3D dimensions. However, this effect was not detectable in the sample with GR, which is a 2D filler, due to the inefficient mixing that results from melt compounding, which does not allow good dispersion and produces a folded and deformed morphology in the filler [4]. This is possible to see in the results of the morphological analysis. At higher filler loadings, the extent of improvement in the mechanical properties might be limited or have a detrimental effect due to the high viscosity of the composite, lower degree of filler dispersion and the higher chance of generating void defects [31]. A different result was observed with GR, which showed a small improvement in the tensile elastic modulus while the flexural one showed greater improvement. This can probably be ascribed to the orientation of the sheets obtained by injection molding. A single GR sheet has an ideal elastic modulus of about 1 TPa and it is one of the strongest materials ever measured (on a micrometric scale). Nonetheless, when these sheets are dispersed in a matrix, they tend to assume a wrinkled aspect rather than a stretched configuration and during GR particles’ restacking they may coalesce and consequently their exfoliation fails. All these phenomena are related to an overall reduction in the aspect ratio and can be assumed to be the main factors responsible for the elastic modulus outcomes [18].

#### 3.2.2. Tensile Strength

**Figure 8:**
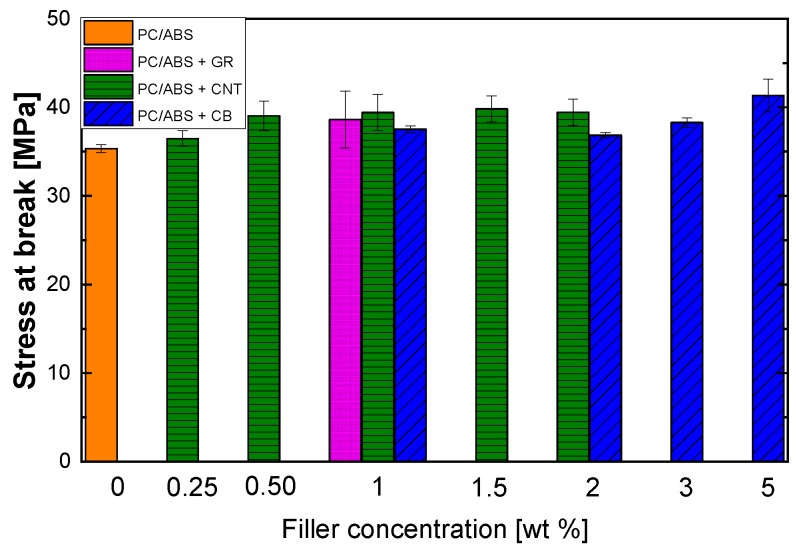
Stress at break of composites obtained by melt blending at different fillers concentrations.

The stress at break increased with increasing filler wt. %, as shown in Figure 8.

The trend related to the stress at break of the PC/ABS composite as a function of CNTs wt.% was similar to that of the elastic modulus, but without significant variation with respect to the unfilled reference matrix. However, this type of composite usually displays a significant reduction in the stress at break due to the absence of a specific interaction between the filler and the matrix. In this case, this property was not negatively altered, even in the presence of a relative high amount of filler. Such results are probably due to the fact that CNTs are preferentially located in the extensible PC phase (as is further discussed in Section 3.3), thus, they do not act as an obstacle during the deformation. Then, the increase in the stress at break is due to the mitigation of the effect of the dispersed particles, which allows the matrix to be deformed without a premature break [32]. The stress at break of PC/ABS with 0.5 wt.% of CNTs increased by 10%. These results suggest a positive stress transfer from the matrix to the fillers as a consequence of the higher surface of interaction between them.

#### 3.2.3. Impact Strength

**Figure 9:**
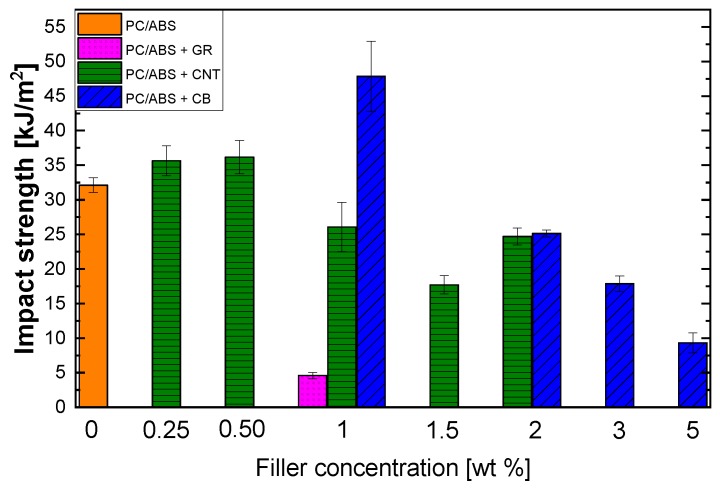
Impact strength of composites obtained by melt blending at different fillers concentrations.

Improvements in tensile strength and modulus are coupled with a reduction in the strain at break, such as those shown for the impact strength test (see Figure 9), which indicates a decrease in polymer toughness and flexibility.

Comparing the results for the impact strength, it is clear that graphene induces fragility in the corresponding composite and a remarkable decrease in the impact strength. This behavior may be ascribed to the poor dispersion of GR and weak interfacial adhesion with the matrix, which results in the absence of a mechanical interlocking effect and introduces defects into the polymer matrix that have a negative effect on the bulk mechanical properties. For the other fillers, the general effect is a slight to moderate increase in the impact strength at low levels of loading, which disappear when the content is higher than 0.5–1 wt. %, and eventually the impact strength decreases. Significant increases in fracture toughness have been reported when 1D nanoparticles [33], such as CNTs, are used as reinforcement for matrix-dominated composites. This increase is linked to the extraordinarily high interface area of 1D nanoparticles and the bridging mechanism that suppresses the propagation of cracks and positively contributes to increased fracture toughness. This is very different for 3D fillers, such as the CB employed in this work. Crack pinning, crack deflection, and plastic void growth are cited by Saghafi et al. [33] as the key mechanisms leading to the increase in fracture toughness for 3D composites. These issues are caused by the obstruction of crack propagation by agglomerated carbon particles. It can be noted that the decrease in impact strength for CB and CNTs is reached at the electrical percolation threshold concentration. Such conditions lead to structural changes in the composite due to the formation of a three-dimensional network. With the addition of more filler, agglomerates of CNTs or CB start to form in the PC/ABS matrix, which leads to the introduction of defects that are responsible for the decrease of the impact strength [34]. Another possible explanation is related to the findings of Ganß et al. [35] for polypropylene composites. They ascribe the decline in impact performance to the increase in filler network density and the consequent minor matrix deformability, which causes stress intensification around the CNTs network. Thus, the strain localization ultimately causes matrix cracking due to severe modulus mismatch between the polymer and filler.

### 3.3. Morphological Characterization

First it was necessary to make a preliminary analysis of the PC/ABS matrix morphology, and then TEM analysis was used to determine the dispersion of the filler particles within the matrix.

**Figure 10:**
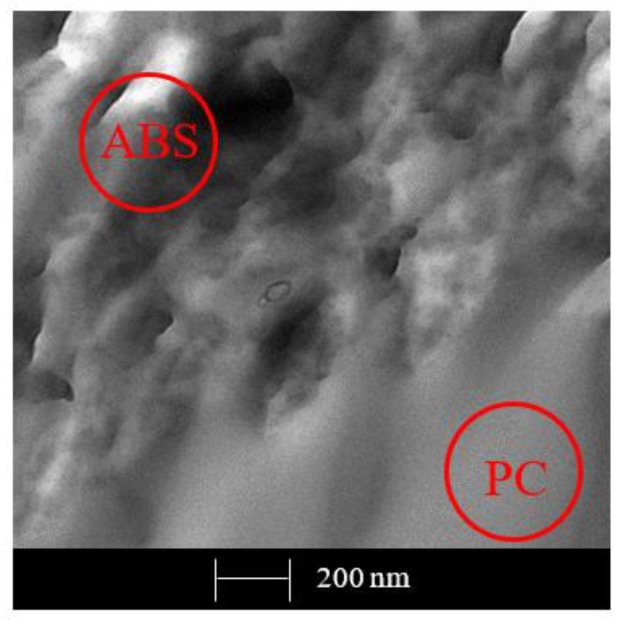
TEM micrograph of pristine PC/ABS blend.

Figure 10 shows the location of the blend’s PC and ABS components. The smooth, lower moiety may be attributed to PC and the rough one to ABS; also some isolated rubbery domains of butadiene are visible within the ABS portion.

**Figure 11:**
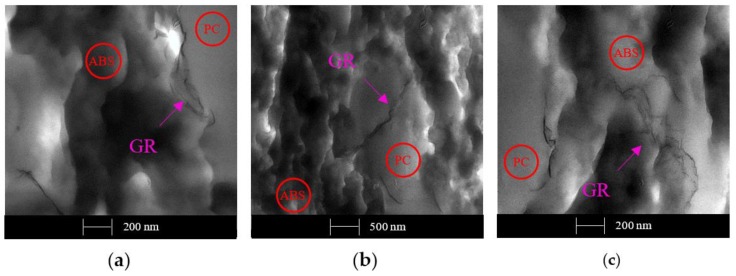
TEM micrographs of a sample with 1 wt. % of GR obtained by the melt extrusion method. Different locations of GR sheets on the PC/ABS blend domains: (**a**) GR located on the surface between PC and ABS domains; (**b**) GR located on PC moiety; and (**c**) GR located on ABS moiety.

Figure 11 shows the GR dispersion on the 1 wt. % sample obtained by the extrusion method.

In Figure 11a, GR sheets are located on the surface between the two polymer domains, while Figure 11b,c show that the GR is inside the PC and ABS, respectively. So, we can assert that GR dispersion by melt extrusion does not have a preferential distribution. However, 1 wt. % of GR does not show the formation of a continuous network, justifying the previous bad conductivity results. 

**Figure 12:**
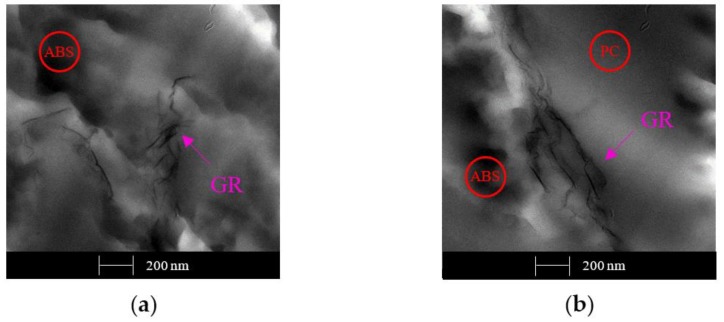
TEM micrographs of samples with 1 wt. % of GR obtained by solution dispersion methods: (**a**) with solvent removal by evaporation; (**b**) with solvent removal by precipitation.

Figure 12 shows the differences between the two GR solution dispersion methods: by evaporation (in Figure 12a) and by precipitation (in Figure 12b) and comparing samples at a reference concentration of 1 wt. %. These micrographs depict a broader path compared to the previous ones, that is, 1 wt. % of GR solution obtained by dispersion has a more continuous network compared to the network obtained by melt extrusion, which is reflected in better electrical conductivity.

**Figure 13:**
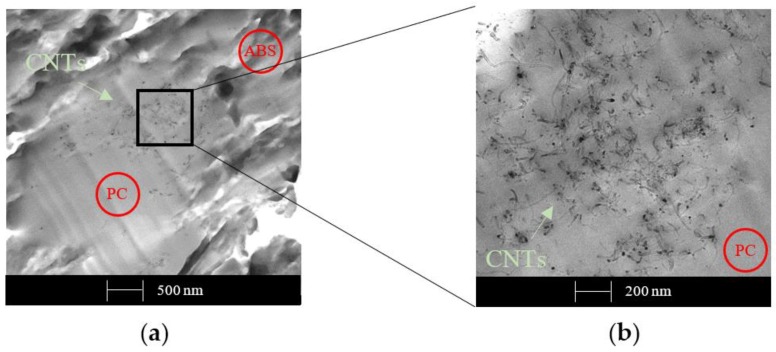
TEM micrographs of sample with 1 wt. % of CNTs obtained by melt extrusion method.

The dispersion of 1 wt. % of CNTs is displayed in Figure 13.

As already asserted in our previous study and by Monemian et al. [36,37], Figure 13a clearly shows that CNTs are placed preferentially in the PC smooth phase; in contrast, the ABS phase seems to be free of particles. The zoomed image (Figure 13b) shows excellent CNTs dispersion within the PC portion. The preferential localization of a filler in one of the two phases is usually explained by the differences in the interfacial energies between the filler and the polymers, which is caused by the different polarities and surface energies.

**Figure 14:**
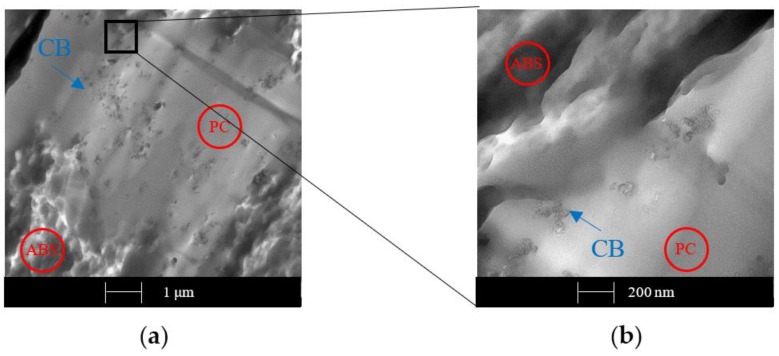
TEM micrographs of sample with 1 wt. % of CB obtained by the melt extrusion method.

Also, 1 wt. % of CB shows segregation on PC moiety, as is observable in Figure 14.

In contrast with the CNTs sample, the zoomed image of the CB sample (Figure 14b) does not show a constant filler network and the 1 wt. % sample does not reach the percolation threshold. These findings are in agreement with the discussion above regarding electrical conductivity: the formation of a continuous path is more difficult with spherical particles such as CB, as Figure 13 and Figure 14 demonstrate, thus, the percolation limit can only be reached at higher filler concentrations.

### 3.4. Reological Characterization

**Figure 15:**
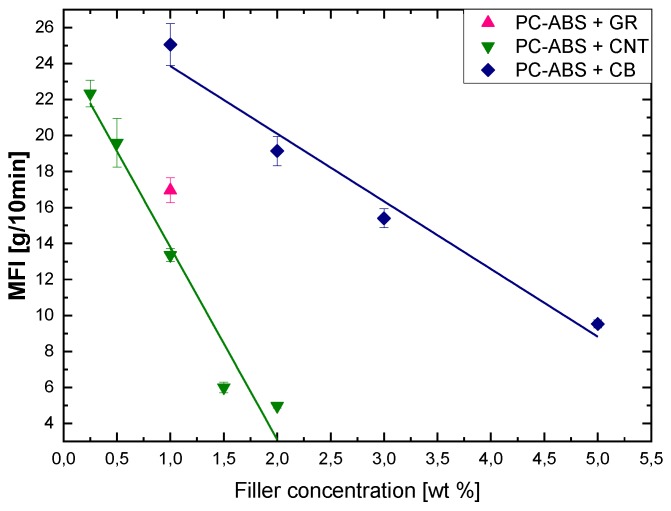
Effect of filler concentration on the melt flow index (MFI) of the PC/ABS composites.

The MFI was evaluated to obtain an indication of the change in viscosity with the particle loadings, as it is an empirical index that is roughly proportional to the reciprocal of the melt viscosity [38]. The MFI variations with different filler concentrations are reported in Figure 15.

The general trend shows a linear decrease with increasing filler loading as a consequence of the higher viscosity of the melt in the presence of the fillers, with a steeper decrease in the case of CNTs compared to CB. With the addition of CNTs and CB, particle–particle interactions increase, and the polymer chains are more restrained. As a result, the viscosity of the composite melt increases. This is exacerbated at high-volume fractions of filler, and when the filler particles are smaller, especially for nano-sized fillers such as CNTs [39]. Interestingly, when the is 1%, CNTs present the lowest MFI, followed by GR and CB, probably because of the high aspect ratio of the filler and better dispersion in the matrix, as confirmed by the volume resistivity measurements. These drastic increases in viscosity are not completely detrimental from a processing point of view since good results can be obtained at very low CNTs content where the MFI is very close to that of the pure matrix. As a consequence, the common practice of using melt mixing seems more suitable for obtaining reinforced polymer composites by using CNTs as filler because this combines good processing with improved electrical and mechanical performance. 

## 4. Conclusions

Carbon black, carbon nanotubes and graphene composite blends were realized by melt blending, using a 0.25%, 0.5%, 1%, 1.5% and 2% filler weight content for CNTs, 1%, 2%, 3%, 5% for CB and 1 wt. % for GR. The electrical resistivity characterization determined a percolation threshold of 0.25% for nanotubes and 3% for carbon black, while no percolation was detected for graphene up to 1% of weight content. For the latter, solution blending was found to be more suitable to obtain better dispersion, and electrical percolation was obtained with a graphene content ranging from 0.5%to 1% by weight.

From the dielectric analysis it was possible to observe a progressive increase in the loss factor with the amount of filler in the composites above the glass transition temperature. An abrupt increase in the loss factor is obtained with filler amounts higher than the percolation threshold due to an interfacial polarization effect.

The mechanical properties of injection molded specimens showed an increase in the elastic modulus with the carbon nanotubes and carbon black content, while there was no significant difference with 1 wt. % of graphene. On the other hand, the latter induced a ductile to fragile transition of the material with a remarkable decrease in the impact strength.

TEM analysis was used to determine the dispersion of filler particles within the matrix. The main difference is related to the dispersion of graphene and the other carbon-based fillers. While carbon black and carbon nanotubes show a preferential dispersion in the PC moiety, graphene is dispersed in both the polymeric phases. Despite this, the folded disposition of the graphene sheets blocks the formation of an electrically conductive network. The graphene filler did not achieve the desired results, although the solvent blending method resulted in a decrease in volumetric resistivity, however, this is actually not economically viable.

In conclusion, the results of our comparison of three carbon-based fillers suggest that CNTs are the best filler choice in terms of electrical and mechanical performance. Compared to the other fillers, CNTs obtained the lowest percolation threshold and the highest loss factor values with regard to electrical properties, moreover, from a mechanical point of view, CNTs showed the best improvement in elastic moduli without decreasing the toughness of the polymer matrix.
